# Association Between Perceived Food Environment and Self-Efficacy for Fruit and Vegetable Consumption Among US Adults, 2007

**Published:** 2011-12-15

**Authors:** Temitope O. Erinosho, April Y. Oh, Richard P. Moser, Linda C. Nebeling, Kia L. Davis, Amy L. Yaroch

**Affiliations:** The Department of Nutrition and Center for Health Promotion and Disease Prevention, University of North Carolina at Chapel Hill. Dr Erinosho was a Cancer Research Training Award (CRTA) postdoctoral fellow at the US National Cancer Institute in Rockville, Maryland, at the time of this work; National Cancer Institute, Rockville, Maryland; National Cancer Institute, Rockville, Maryland; National Cancer Institute, Rockville, Maryland; Harvard School of Public Health, Boston, Massachusetts. Kia L. Davis was a contractor with SAIC-Frederick, Inc, Rockville, Maryland, at the time of this work; Gretchen Swanson Center for Nutrition, Omaha, Nebraska

## Abstract

Consumption of diets high in fruits and vegetables is associated with reduced risk of chronic diseases, and self-efficacy and the food environment influence consumption of fruits and vegetables. We analyzed data from 3,021 non-Hispanic white (n = 2,187) and non-Hispanic black (n = 834) US adults who responded to National Cancer Institute's 2007 Food Attitudes and Behaviors Survey to assesss self-efficacy and perception of the food environment. Adults who perceived that it was easy to obtain fruits and vegetables when they ate out reported greater self-efficacy to consume fruits and vegetables than did participants who did not have this perception (odds ratio [OR] = 1.56, 95% confidence interval [CI], 1.24-1.97). However, adults who perceived that fruits were not available at restaurants where they ate out (OR = 0.65, 95% CI, 0.50-0.86) or that other (ie, non–fast food) restaurants offered enough choices of fruits and vegetables on their menus (OR = 0.76, 95% CI, 0.61-0.97) reported lower self-efficacy to consume fruits and vegetables than did participants who did not have these perceptions. Findings suggest that perceptions about availability of fruits and vegetables in restaurants are important to promote self-efficacy for consuming fruits and vegetables among adults.

## Introduction

Consumption of diets high in fruits and vegetables (F/V) is associated with reduced risk of chronic diseases (1). Self-efficacy, an individual's confidence to perform certain behaviors (2), is associated with increased consumption of F/V (3). The food environment, particularly neighborhood availability and access to healthful food stores and restaurants, influences fruit and vegetable consumption (4,5). Research examining perceptions of the food environment in relation to adults' self-efficacy to consume F/V is limited. According to social cognitive theory, a person's behavior, personal characteristics, and environments continuously interact with and influence one another (2,6). The purpose of this study was to evaluate the association between adults' perceptions of their neighborhood and restaurant food environment and their self-efficacy to eat F/V.

## Methods

We analyzed data from the US National Cancer Institute's Food Attitudes and Behaviors (FAB) Survey, a panel survey that was administered by mail to adults in the United States in 2007. The FAB survey collected data about consumption of F/V and the attitudes and perceptions associated with fruit and vegetable consumption. Detailed descriptions of the development and initial evaluation of the FAB Survey items can be found elsewhere (7,8). Briefly, participants were selected from Synovate Global Opinion Panels (Chicago, Illinois) through stratified random sampling. The FAB Survey was mailed to 5,803 adults; 3,418 surveys were returned, yielding a response rate of 59%. The final FAB Survey sample consisted of 3,397 adults. For this study, we limited our sample to non-Hispanic white (n = 2,187) and non-Hispanic black (n = 834) adults because of the small sample of "other" racial/ethnic groups (n = 291) and participants who did not report their race/ethnicity (n = 85), resulting in a final sample of 3,021 adults.

Self-efficacy to consume F/V, defined as confidence in one's ability to consume F/V, was measured by using 5 items that asked participants to indicate how confident they were that they could consume F/V in various situations. Participants were asked, "How confident are you that you could . . . " and were given the following 5 scenarios: 1) Eat a healthy snack, like a fruit or a vegetable, when you're really hungry?, 2) Eat healthy foods, like fruits or vegetables, when you are tired?, 3) Eat fruit instead of cake, cookies, candy, ice cream, or other sweets for dessert?, 4) Eat fruits and vegetables when your family and friends are eating junk foods like chips, cookies, or candy?, and 5) Buy or bring fruits and vegetables to eat at work? Responses were on a 5-point Likert-type scale, 1 being "not at all confident" to 5 being "very confident." A scale score was calculated by taking the mean of the 5 items (Cronbach α = 0.85). For ease of interpretation, a dichotomous final summary score, using a median split (median = 3.8), was created for all subsequent analyses, given that the results reported here did not change when using a continuous outcome. Participants with median self-efficacy scores of 3.80 or more were categorized as having "higher self-efficacy" to consume F/V, and those with median self-efficacy scores of 3.80 or less were categorized as having "lower self-efficacy" to consume F/V.

Perceptions of the neighborhood and restaurant food environment were assessed by using 6 statements that asked participants about the ease of purchasing F/V in neighborhoods and while eating out and about the availability of F/V at fast food and other restaurants (ie, non–fast food). Responses were on a 5-point Likert-type scale, ranging from 1, being "strongly disagree" to 5, being "strongly agree." The statements were "It is hard for me to purchase fruits and vegetables in my neighborhood," "When I eat out, it is easy for me to get fruits and vegetables," "Fast food places offer enough choices of fruits and vegetables on their menus," "Other restaurants offer enough choices of fruits and vegetables on their menus," "The restaurants I go to don't serve fruit," and "The restaurants I go to don't serve vegetables." Participants' responses were coded as *disagree* (by combining "strongly disagree" and "somewhat disagree"), *neutral* (by using the middle option), and *agree* (by combining "strongly agree" and "somewhat agree").

We used SAS version 9.1 (SAS Inc, Cary, North Carolina) to calculate sociodemographic characteristics, assess perceptions of the food environment, and test for bivariate associations. We used binary logistic regression to determine the association between perceptions of the food environment and self-efficacy to consume F/V, controlling for sociodemographic characteristics. Statistical significance was set at *P* < .05. All analyses were weighted with sampling weights that were poststratified by sex, race/ethnicity, age, education level, and annual household income using the 2000 US Census estimates.

## Results

Most participants reported that they did not find it hard to purchase F/V in their neighborhood (77%) (Table 1). Approximately 40% of participants reported that obtaining F/V when they ate out was easy. More participants reported that other (ie, non–fast food) restaurants offered enough choices of F/V on their menu (44%) compared with fast food restaurants (13%).

Participants who perceived that it was easy to obtain F/V when they ate out had higher self-efficacy to consume F/V than did participants who did not have this perception (Table 2). However, participants who perceived that other (ie, non–fast food) restaurants offered enough choices of F/V on their menus or who perceived that fruits were not available at restaurants where they ate had lower self-efficacy to consume F/V than did participants who did not have these perceptions. No associations were found between perceived access to F/V in neighborhoods and fast food restaurants and self-efficacy to consume F/V.

## Discussion

We evaluated the association between perceptions of the neighborhood and restaurant food environment and self-efficacy to consume F/V among US adults. Results indicated that adults who reported higher self-efficacy to consume F/V also tended to perceive that it was easy for them to get F/V when they ate out and that fruits were available at restaurants where they ate. Unexpectedly, our results show that adults who perceived that other restaurants offered enough choices of F/V on their menus reported lower self-efficacy to consume F/V.

To explore this finding further, we examined characteristics of people who reported that other restaurants offer enough choices of F/V on menus. We found that these participants were mostly non-Hispanic white, were aged 35 to 54, earned less than $25,000, and were more likely to report lower self-efficacy to consume F/V overall. Because a large proportion of these participants were low-income, they may tend to eat mostly at fast food restaurants; although they may perceive that F/V are available at other (ie, non–fast food) restaurants, they may not be eating at those restaurants.

In this study, perceived access to F/V in neighborhoods and fast food restaurants was not associated with self-efficacy to consume F/V. Prior studies found that self-efficacy is associated with higher fruit and vegetable intake (3). Other studies also report that easy access to F/V in neighborhoods, food stores, and restaurants is associated with higher fruit and vegetable intake (3,4). However, on the basis of cross-sectional data, this is the first study to our knowledge that used a national sample to examine perceptions of the neighborhood and restaurant food environment in relation to self-efficacy to consume F/V.

Our study has limitations. We used self-reported data and only analyzed the data of non-Hispanic whites and African Americans. Because the sample was drawn from a consumer opinion panel, the weighted analyses only allow us to generalize to people in the panel. However, data from this panel had prevalence estimates similar to those of the Behavioral Risk Factor Surveillance System, which uses a random-digit-dialed approach (9). Furthermore, measures of the neighborhood food environment were obtained on the basis of a single item that asked participants whether it was hard to purchase F/V in their neighborhood. Perceived access to purchasing F/V is only 1 aspect of the food purchasing environment; other measures may include perceptions of food quality and cost. In contrast, 5 items were used to measure perceptions about restaurant availability of F/V. A strength of the study was the oversampling of African Americans during recruitment to increase precision estimates.

Our findings suggest that perceived availability of F/V in restaurants is an area in which adults' self-efficacy for consuming F/V can be promoted, which potentially could result in increased fruit and vegetable intake among adults. Health promotion efforts should focus on encouraging restaurants to offer F/V on their menus and to promote consumption of F/V among their adult patrons.

## Figures and Tables

**Table 1:** Sociodemographic Characteristics, Perceptions of the Neighborhood and Restaurant Food Environment, and Reported Self-Efficacy to Consume Fruits and Vegetables Among Adults (N = 3,021), 2007 Food Attitudes and Behaviors Survey^a^

**Variable**	n (%)
**Sociodemographic Characteristics**	
**Sex**	
Female	1,828 (53)
Male	1,175 (47)
**Age, y**	
18-34	840 (30)
35-54	1,196 (36)
≥55	972 (34)
**Race/ethnicity**	
Non-Hispanic white	2,187 (87)
Non-Hispanic black	834 (13)
**Education**	
Less than high school degree	355 (13)
High school degree	932 (32)
Some college	897 (29)
College degree or higher	823 (26)
**Income, $**	
<25,000	767 (28)
25,000-49,999	788 (29)
50,000-74,999	562 (17)
≥75,000	904 (26)
**Geographic region of residence**	
Northeast	603 (20)
Midwest	677 (24)
South	1,248 (39)
West	493 (18)
**Perceptions of the Neighborhood and Restaurant Food Environment**	
**Hard to purchase fruits and vegetables in my neighborhood**	
Disagree	2,289 (77)
Neutral	341 (12)
Agree	316 (11)
**Easy to get fruits and vegetables when I eat out**	
Disagree	813 (29)
Neutral	893 (31)
Agree	1,234 (40)
**Fast food places offer enough choices of fruits and vegetables on their menu**	
Disagree	1,975 (66)
Neutral	576 (20)
Agree	387 (13)
**Other (ie, non–fast food) restaurants offer enough choices of fruits and vegetables on their menu**	
Disagree	785 (27)
Neutral	828 (28)
Agree	1,335 (44)
**Restaurants I go to don't serve fruit**	
Disagree	1,787 (59)
Neutral	664 (23)
Agree	518 (18)
**Restaurants I go to don't serve vegetables**	
Disagree	2,238 (74)
Neutral	517 (19)
Agree	210 (7)
**Self-Efficacy to Consume Fruits and Vegetables**	
Lower self-efficacy to consume fruits and vegetables (median self-efficacy score <3.80)	1,810 (54)
Higher self-efficacy to consume fruits and vegetables (median self-efficacy score ≥3.80)	1,516 (46)

a Frequencies represent the actual number of participants; percentages were weighted by sex, race/ethnicity, age, education level, and annual household income based on 2000 US Census estimates.

**Table 2:** Associations Between Perceptions of Food Environment and Reported Self-Efficacy to Consume Fruits and Vegetables (F/V) Among US Adults, 2007 Food Attitudes and Behaviors Survey^a^^,^ b

**Variable**	Self-Efficacy to Consume F/V, OR (95% CI)	*P* Value
**Hard to purchase F/V in my neighborhood**		
Disagree	1 [Reference]	
Neutral	0.97 (0.73-1.29)	.85
Agree	1.15 (0.86-1.53)	.34
**Easy to get F/V when I eat out**		
Disagree	1 [Reference]	
Neutral	1.02 (0.81-1.29)	.88
Agree	1.56 (1.24-1.97)	<.001
**Fast food places offer enough choices of F/V on their menu**		
Disagree	1 [Reference]	
Neutral	0.89 (0.70-1.12)	.30
Agree	0.85 (0.65-1.12)	.26
**Other restaurants (non–fast food) offer enough choices of F/V on their menu**		
Disagree	1 [Reference]	
Neutral	0.73 (0.57-0.94)	.01
Agree	0.76 (0.61-0.97)	.02
**Restaurants I go to don't serve fruit**		
Disagree	1 [Reference]	
Neutral	0.79 (0.61-1.01)	.06
Agree	0.65 (0.50-0.86)	<.001
**Restaurants I go to don't serve vegetables**		
Disagree	1 [Reference]	
Neutral	0.90 (0.68-1.19)	0.45
Agree	1.04 (0.70-1.56)	0.83
**Sex**		
Male	1 [Reference]	
Female	1.35 (1.14-1.61)	<.001
**Age, y**		
18-34	1 [Reference]	
35-54	0.92 (0.74-1.13)	0.41
55 or older	1.29 (1.04-1.62)	0.02
**Race/ethnicity**		
Non-Hispanic white	1 [Reference]	
Non-Hispanic black	1.58 (1.30-1.93)	<.001
**Income, $**		
<25,000	1 [Reference]	
25,000-49,999	1.10 (0.85-1.41)	0.47
50,000-74,999	1.24 (0.95-1.63)	0.12
≥75,000	1.50 (1.17-1.92)	<.001
**Geographic region of residence**		
Northeast	1 [Reference]	
Midwest	0.99 (0.76-1.28)	0.92
South	1.13 (0.89-1.43)	0.32
West	0.99 (0.75-1.31)	0.96

Abbreviation: OR, odds ratio; CI, confidence interval.

a Sampling weights, poststratified by sex, race/ethnicity, age, education level, and annual household income, using 2000 US Census estimates, were applied to the regression model.

b The 6 items that measured perceptions of the neighborhood and restaurant food environment were kept in the same regression model because a factor analysis showed that the items operated independently of each other (Cronbach α = 0.28; variance inflation factor, 1.1-1.6; tolerance, 0.6-0.9).
